# An improvement of the Lyapunov inequality for certain higher order differential equations

**DOI:** 10.1186/s13660-018-1809-5

**Published:** 2018-08-17

**Authors:** Haidong Liu

**Affiliations:** School of Mathematical Sciences, Qufu Normal University, Qufu, P.R. China

**Keywords:** Lyapunov inequality, Half-linear differential equation, Sobolev inequality, Riemann zeta function

## Abstract

In this paper, we establish two Lyapunov inequalities for some half-linear higher order differential equations with anti-periodic boundary conditions. Our result improves that obtained by Wang (Appl. Math. Lett. 25:2375–2380, 2012).

## Introduction

In 1907, Lyapunov [2] proved the following remarkable result known as Lyapunov inequality. If *u* is a solution of

1.1 $$ u''+q(t)u=0, $$

satisfying $u(a)=u(b)=0$ ($a< b$) and $u\neq 0$, then

$$ \int_{a}^{b} \bigl\vert q(t) \bigr\vert \, \mathrm{d}t>\frac{4}{b-a}. $$

Since then, Lyapunov inequality and many of its generalizations have gained a great deal of attention (see [3–12] and the references therein), because these results have found many applications in the study of various properties of solutions of differential and difference equations such as oscillation theory, disconjugacy, and eigenvalue problems.

In the last twenty years, a lot of efforts have been made to obtain similar results for higher order differential equations (see [1, 13–17] and the references therein), and other type integral inequalities (see [18–30] and the references therein). In particular, Çakmak [13] considered the following even higher order linear differential equation:

1.2 $$ u^{(2m)}(t)+r(t)u(t)=0,\quad t\in [a,b], $$

where $r\in C([a,b],[0,\infty))$ and *u* satisfies the following boundary conditions:

1.3 $$ u^{(2i)}(a)=u^{(2i)}(b)=0, \quad i=0,1,2,\ldots,m-1, $$

and he obtained the following result. If there exists a nontrivial solution *u* of Eq. (1.2) satisfying (1.3), then one has

1.4 $$ \int_{a}^{b}r(t)\,\mathrm{d}t>\frac{2^{2m}}{(b-a)^{2m-1}}. $$

Later, Watanabe et al. [14] used a Sobolev inequality to get a new Lyapunov inequality for Eq. (1.2)

$$ \int_{a}^{b}r(t)\,\mathrm{d}t>\frac{2^{2m}}{(b-a)^{2m-1}}\cdot \frac{ \pi^{2m}}{{2(2^{2m}-1)\zeta (2m)}}, $$

where

$$ \zeta (s)=\sum_{n=1}^{+\infty }\frac{1}{n^{s}}, \qquad \operatorname{Re}(s)>1, $$

is the Riemann zeta function. Their result sharpened the result of Çakmak [13].

Recently, Wang [1] considered the following $(m+1)$-order half-linear differential equation:

1.5 $$ \bigl( \bigl\vert u^{(m)}(t) \bigr\vert ^{p-2}u^{(m)}(t) \bigr)'+r(t) \bigl\vert u(t) \bigr\vert ^{p-2}u(t)=0, \quad t\in (a,b), $$

where $r\in C([a,b],\mathbb{R})$, $m\in \mathbb{N}$, $p>1$ is a constant, and *u* satisfies the following anti-periodic boundary conditions:

1.6 $$ u^{(i)}(a)+u^{(i)}(b)=0,\quad i=0,1,2,\ldots,m, $$

and he obtained the following result. If there exists a nontrivial solution *u* of Eq. (1.5) satisfying (1.6), then

1.7 $$ \int_{a}^{b} \bigl\vert r(t) \bigr\vert \, \mathrm{d}t>2 \biggl( \frac{2}{b-a} \biggr) ^{m(p-1)}. $$

In the present paper, we shall use the Sobolev inequality established in [14] to establish two Lyapunov inequalities for Eq. (1.5) with the anti-periodic boundary conditions (1.6). Our result improves that obtained by Wang [1].

## Main results

### Lemma 2.1

([14])

*For* $m\geq 1$, *define the following Sobolev space*:

$$ H_{m}=\bigl\{ u|u^{(m)}\in L^{2}[a,b], u^{(k)}(a)+u^{(k)}(b)=0, k=0,1,2, \ldots,m-1\bigr\} . $$

*There exists a positive constant* $C_{m}$ *such that*, *for any* $u\in H_{m}$, *the Sobolev inequality*

$$ \Bigl(\sup_{a\leq t\leq b} \bigl\vert u(t) \bigr\vert \Bigr)^{2}\leq C_{m} \int_{a}^{b} \bigl\vert u^{(m)}(t) \bigr\vert ^{2} \,\mathrm{d}t $$

*holds*, *where*

$$ C_{m}=\frac{2(2^{2m}-1)(b-a)^{2m-1}\zeta (2m)}{2^{2m}\pi^{2m}},\quad m=1,2, \ldots, $$

*and the constants* $\{C_{m}\}$ *are sharp*,

$$ \zeta (s)=\sum_{n=1}^{+\infty }\frac{1}{n^{s}}, \qquad \operatorname{Re}(s)>1, $$

*is the Riemann zeta function*.

### Remark 2.1

From the definition of $H_{m}$, we can easily get if $u\in H_{m}$, then $u^{(m-1)}\in H_{1}$.

### Lemma 2.2

*If* *u* *is a nontrivial solution of Eq*. (1.5) *satisfying the anti*-*periodic boundary conditions* (1.6), *then the inequalities*

2.1 $$ \bigl\vert u(t) \bigr\vert \leq \frac{(b-a)^{m-1}}{2^{m}} \int_{a}^{b} \bigl\vert u^{(m)}(s) \bigr\vert \,\mathrm{d}s, \quad t\in [a,b], $$

*and*

2.2 $$ \bigl\vert u^{(m-1)}(t) \bigr\vert \leq \frac{1}{2} \int_{a}^{b} \bigl\vert u^{(m)}(s) \bigr\vert \,\mathrm{d}s, \quad t\in [a,b] $$

*hold*.

### Proof

Since the nontrivial solution *u* of Eq. (1.5) satisfies the anti-periodic boundary conditions (1.6), then we have

$$ u(t)=\frac{1}{2} \int_{a}^{t}u'(s)\,\mathrm{d}s- \frac{1}{2} \int_{t}^{b}u'(s) \,\mathrm{d}s, \quad t\in [a,b]. $$

So,

2.3 $$ \bigl\vert u(t) \bigr\vert \leq \frac{1}{2} \int_{a}^{t} \bigl\vert u'(s) \bigr\vert \,\mathrm{d}s+\frac{1}{2} \int _{t}^{b} \bigl\vert u'(s) \bigr\vert \,\mathrm{d}s=\frac{1}{2} \int_{a}^{b} \bigl\vert u'(s) \bigr\vert \,\mathrm{d}s, \quad t\in [a,b]. $$

Similarly, we have

2.4 $$ \bigl\vert u^{(k)}(t) \bigr\vert \leq \frac{1}{2} \int_{a}^{b} \bigl\vert u^{(k+1)}(s) \bigr\vert \,\mathrm{d}s, \quad t\in [a,b], k=1,2,\ldots, m-1, $$

then (2.2) holds. It follows from (2.4) that

2.5 $$ \int_{a}^{b} \bigl\vert u^{(k)}(s) \bigr\vert \,\mathrm{d}s\leq \frac{b-a}{2} \int_{a}^{b} \bigl\vert u ^{(k+1)}(s) \bigr\vert \,\mathrm{d}s, \quad k=1,2,\ldots, m-1. $$

From (2.3) and (2.5), we obtain

2.6 $$ \bigl\vert u(t) \bigr\vert \leq \frac{b-a}{2^{2}} \int_{a}^{b} \bigl\vert u''(s) \bigr\vert \,\mathrm{d}s\leq \cdots \leq \frac{(b-a)^{m-1}}{2^{m}} \int_{a}^{b} \bigl\vert u^{(m)}(s) \bigr\vert \,\mathrm{d}s, \quad t\in [a,b], $$

i.e., (2.1) holds. □

### Theorem 2.1

*If* *u* *is a nontrivial solution of Eq*. (1.5) *satisfying the anti*-*periodic boundary conditions* (1.6), *then the inequality*

2.7 $$ \int_{a}^{b} \bigl\vert r(t) \bigr\vert \, \mathrm{d}t>2 \biggl( \frac{2}{b-a} \biggr) ^{m(p-1)} \frac{ \pi^{m(p-1)}}{2^{(p-1)/2}(2^{2m}-1)^{(p-1)/2}(\zeta (2m))^{(p-1)/2}} $$

*holds*, *where* $p>2$.

### Proof

Since the nontrivial solution *u* of Eq. (1.5) satisfies the anti-periodic boundary conditions (1.6), it is easy to see that *u* is an element of $H_{m}$. Multiplying (1.5) by $u^{(m-1)}(t)$ and integrating over $[a, b]$, yields

2.8 $$ \int_{a}^{b}\bigl( \bigl\vert u^{(m)}(t) \bigr\vert ^{p-2}u^{(m)}(t)\bigr)'u^{(m-1)}(t) \,\mathrm{d}t+ \int_{a}^{b}r(t) \bigl\vert u(t) \bigr\vert ^{p-2}u(t)u^{(m-1)}(t)\,\mathrm{d}t=0. $$

Using integration by parts to the first integral on the left-hand side of (2.8) and the anti-periodic boundary conditions (1.6), we have

$$ \int_{a}^{b} \bigl\vert u^{(m)}(t) \bigr\vert ^{p}\,\mathrm{d}t= \int_{a}^{b}r(t) \bigl\vert u(t) \bigr\vert ^{p-2}u(t)u ^{(m-1)}(t)\,\mathrm{d}t. $$

So

2.9 $$\begin{aligned} \int_{a}^{b} \bigl\vert u^{(m)}(t) \bigr\vert ^{p}\,\mathrm{d}t =& \int_{a}^{b}r(t) \bigl\vert u(t) \bigr\vert ^{p-2}u(t)u ^{(m-1)}(t)\,\mathrm{d}t \\ \leq & \int_{a}^{b} \bigl\vert r(t) \bigr\vert \bigl\vert u(t) \bigr\vert ^{p-1} \bigl\vert u^{(m-1)}(t) \bigr\vert \,\mathrm{d}t \\ \leq & \Bigl(\sup_{a\leq t\leq b} \bigl\vert u(t) \bigr\vert \Bigr)^{p-1}\sup_{a\leq t\leq b} \bigl\vert u ^{(m-1)}(t) \bigr\vert \int_{a}^{b} \bigl\vert r(t) \bigr\vert \, \mathrm{d}t. \end{aligned}$$

By Lemma 2.1 and Remark 2.1, we obtain

2.10 $$ \Bigl(\sup_{a\leq t\leq b} \bigl\vert u(t) \bigr\vert \Bigr)^{p-1}\leq C_{m}^{(p-1)/2} \biggl( \int _{a}^{b} \bigl\vert u^{(m)}(t) \bigr\vert ^{2}\,\mathrm{d}t \biggr)^{(p-1)/2} $$

and

2.11 $$ \sup_{a\leq t\leq b} \bigl\vert u^{(m-1)}(t) \bigr\vert \leq C_{1}^{1/2} \biggl( \int_{a}^{b} \bigl\vert u ^{(m)}(t) \bigr\vert ^{2}\,\mathrm{d}t \biggr)^{1/2}. $$

Multiplying (2.10) and (2.11), we have

2.12 $$ \Bigl(\sup_{a\leq t\leq b} \bigl\vert u(t) \bigr\vert \Bigr)^{p-1}\cdot \sup_{a\leq t\leq b} \bigl\vert u ^{(m-1)}(t) \bigr\vert \leq C_{m}^{(p-1)/2}C_{1}^{1/2} \biggl( \int_{a}^{b} \bigl\vert u^{(m)}(t) \bigr\vert ^{2} \,\mathrm{d}t \biggr)^{p/2}. $$

By using Hölder’s inequality

2.13 $$ \int_{a}^{b} \bigl\vert f(t)g(t) \bigr\vert \, \mathrm{d}t\leq \biggl( \int_{a}^{b} \bigl\vert f(t) \bigr\vert ^{ \alpha }\,\mathrm{d}t \biggr) ^{1/\alpha } \biggl( \int_{a}^{b} \bigl\vert g(t) \bigr\vert ^{ \beta }\,\mathrm{d}t \biggr) ^{1/\beta } $$

with $f(t)=1$, $g(t)=|u^{(m)}(t)|^{2}$, $\alpha =\frac{p}{p-2}$, and $\beta =\frac{p}{2}$, we obtain that

$$ \int_{a}^{b} \bigl\vert u^{(m)}(t) \bigr\vert ^{2}\,\mathrm{d}t\leq (b-a)^{(p-2)/p} \biggl( \int _{a}^{b} \bigl\vert u^{(m)}(t) \bigr\vert ^{p}\,\mathrm{d}t \biggr)^{2/p}. $$

Thus

2.14 $$ \biggl( \int_{a}^{b} \bigl\vert u^{(m)}(t) \bigr\vert ^{2}\,\mathrm{d}t \biggr)^{p/2}\leq (b-a)^{(p-2)/2} \int_{a}^{b} \bigl\vert u^{(m)}(t) \bigr\vert ^{p}\,\mathrm{d}t. $$

From (2.12) and (2.14), we have

2.15 $$\begin{aligned}& \Bigl(\sup_{a\leq t\leq b} \bigl\vert u(t) \bigr\vert \Bigr)^{p-1}\cdot \sup_{a\leq t\leq b} \bigl\vert u ^{(m-1)}(t) \bigr\vert \\& \quad \leq C_{m}^{(p-1)/2}C_{1}^{1/2}(b-a)^{(p-2)/2} \int_{a} ^{b} \bigl\vert u^{(m)}(t) \bigr\vert ^{p}\,\mathrm{d}t. \end{aligned}$$

From (2.9) and (2.15), we get

2.16 $$ \int_{a}^{b} \bigl\vert u^{(m)}(t) \bigr\vert ^{p}\,\mathrm{d}t \leq C_{m}^{(p-1)/2}C_{1}^{1/2}(b-a)^{(p-2)/2} \int_{a}^{b} \bigl\vert u^{(m)}(t) \bigr\vert ^{p}\,\mathrm{d}t \int_{a}^{b} \bigl\vert r(t) \bigr\vert \, \mathrm{d}t. $$

Now, we claim that $\int_{a}^{b}|u^{(m)}(t)|^{p}\,\mathrm{d}t>0$. In fact, if the above inequality is not true, we have $\int_{a}^{b}|u^{(m)}(t)|^{p} \,\mathrm{d}t=0$, then $u^{(m)}(t)=0$ for $t\in [a, b]$. By the anti-periodic conditions (1.6), we obtain $u(t)=0$ for $t\in [a, b]$, which contradicts $u(t)\not \equiv 0$, $t\in [a, b]$. Thus dividing both sides of (2.16) by $\int_{a}^{b}|u^{(m)}(t)|^{p}\,\mathrm{d}t$, we obtain the inequality

2.17 $$ \int_{a}^{b} \bigl\vert r(t) \bigr\vert \, \mathrm{d}t\geq \frac{1}{C_{m}^{(p-1)/2}C_{1}^{1/2}(b-a)^{(p-2)/2}}. $$

Since

$$ C_{m}=\frac{2(2^{2m}-1)(b-a)^{2m-1}\zeta (2m)}{2^{2m}\pi^{2m}} \quad \text{and}\quad \zeta (2)= \frac{\pi^{2}}{6}, $$

we get

$$\begin{aligned} \biggl( \frac{1}{C_{m}} \biggr) ^{(p-1)/2} =& \biggl( \frac{2^{2m}\pi^{2m}}{2(2^{2m}-1)(b-a)^{2m-1} \zeta (2m)} \biggr) ^{(p-1)/2} \\ =&\frac{2^{m(p-1)}\pi^{m(p-1)}}{2^{(p-1)/2}(2^{2m}-1)^{(p-1)/2}(b-a)^{ \frac{(2m-1)(p-1)}{2}}(\zeta (2m))^{(p-1)/2}}, \end{aligned}$$

and

$$\begin{aligned} \biggl( \frac{1}{C_{1}} \biggr) ^{(p-1)/2} =& \biggl( \frac{2^{2}\pi^{2}}{2(2^{2}-1)(b-a)^{2-1} \zeta (2)} \biggr) ^{(p-1)/2} \\ =&\frac{2}{(b-a)^{(p-1)/2}}. \end{aligned}$$

Then

2.18 $$\begin{aligned}& \frac{1}{C_{m}^{(p-1)/2}C_{1}^{1/2}(b-a)^{(p-2)/2}} \\& \quad =\frac{2^{m(p-1)}\pi^{m(p-1)}2}{2^{(p-1)/2}(2^{2m}-1)^{(p-1)/2}(b-a)^{m(p-1)}( \zeta (2m))^{(p-1)/2}} \\& \quad =2 \biggl( \frac{2}{b-a} \biggr) ^{m(p-1)}\frac{\pi^{m(p-1)}}{2^{(p-1)/2}(2^{2m}-1)^{(p-1)/2}( \zeta (2m))^{(p-1)/2}}. \end{aligned}$$

So, from (2.17) and (2.18), we get (2.7) holds. Moreover, the inequality in (2.7) is strict since *u* is not a constant. This completes the proof of Theorem 2.1. □

### Theorem 2.2

*If* *u* *is a nontrivial solution of Eq*. (1.5) *satisfying the anti*-*periodic boundary conditions* (1.6), *then the inequality*

2.19 $$ \int_{a}^{b} \bigl\vert r(t) \bigr\vert \, \mathrm{d}t>\frac{2^{m(p-1)+1}}{(b-a)^{m(p-1)}} $$

*holds*, *where* $1< p<2$.

### Proof

As shown in the proof of Theorem 2.1, (2.9) holds, that is,

$$\begin{aligned} \int_{a}^{b} \bigl\vert u^{(m)}(t) \bigr\vert ^{p}\,\mathrm{d}t \leq & \Bigl(\sup_{a\leq t \leq b} \bigl\vert u(t) \bigr\vert \Bigr)^{p-1}\sup_{a\leq t\leq b} \bigl\vert u^{(m-1)}(t) \bigr\vert \int_{a} ^{b} \bigl\vert r(t) \bigr\vert \, \mathrm{d}t. \end{aligned}$$

By using Hölder’s inequality (2.13) with $f(t)=|u^{(m)}(t)|$, $g(t)=1$, $\alpha =p$, and $\beta =\frac{p}{p-1}$, we obtain that

$$ \int_{a}^{b} \bigl\vert u^{(m)}(t) \bigr\vert \,\mathrm{d}t\leq \biggl( \int_{a}^{b} \bigl\vert u^{(m)}(t) \bigr\vert ^{p} \,\mathrm{d}t \biggr)^{1/p}(b-a)^{(p-1)/p}. $$

Thus

2.20 $$ (b-a)^{1-p} \biggl( \int_{a}^{b} \bigl\vert u^{(m)}(t) \bigr\vert \,\mathrm{d}t \biggr)^{p}\leq \int _{a}^{b} \bigl\vert u^{(m)}(t) \bigr\vert ^{p}\,\mathrm{d}t. $$

From (2.1) and (2.2), we have

2.21 $$\begin{aligned}& \Bigl(\sup_{a\leq t\leq b} \bigl\vert u(t) \bigr\vert \Bigr)^{p-1}\sup_{a\leq t\leq b} \bigl\vert u ^{(m-1)}(t) \bigr\vert \\& \quad \leq \frac{(b-a)^{(m-1)(p-1)}}{2^{m(p-1)}} \biggl( \int_{a}^{b} \bigl\vert u^{(m)}(t) \bigr\vert \,\mathrm{d}t \biggr)^{p-1} \frac{1}{2} \int_{a}^{b} \bigl\vert u^{(m)}(t) \bigr\vert \,\mathrm{d}t \\& \quad =\frac{(b-a)^{(m-1)(p-1)}}{2^{m(p-1)+1}} \biggl( \int_{a}^{b} \bigl\vert u^{(m)}(t) \bigr\vert \,\mathrm{d}t \biggr)^{p}. \end{aligned}$$

Using (2.9), (2.20), and (2.21), we get

2.22 $$\begin{aligned}& (b-a)^{1-p} \biggl( \int_{a}^{b} \bigl\vert u^{(m)}(t) \bigr\vert \,\mathrm{d}t \biggr)^{p} \leq \frac{(b-a)^{(m-1)(p-1)}}{2^{m(p-1)+1}} \biggl( \int_{a}^{b} \bigl\vert u ^{(m)}(t) \bigr\vert \,\mathrm{d}t \biggr)^{p} \int_{a}^{b} \bigl\vert r(t) \bigr\vert \, \mathrm{d}t. \end{aligned}$$

Now, we claim that $\int_{a}^{b}|u^{(m)}(t)|\,\mathrm{d}t>0$. In fact, if the above inequality is not true, we have $\int_{a}^{b}|u^{(m)}(t)| \,\mathrm{d}t=0$, then $u^{(m)}(t)=0$ for $t\in [a, b]$. By the anti-periodic conditions (1.6), we obtain $u(t)=0$ for $t\in [a, b]$, which contradicts $u(t)\not \equiv 0$, $t\in [a, b]$. Thus, dividing both sides of (2.22) by $(\int_{a}^{b}|u^{(m)}(t)|\,\mathrm{d}t )^{p}$, we obtain the inequality

2.23 $$ \int_{a}^{b} \bigl\vert r(t) \bigr\vert \, \mathrm{d}t\geq \frac{2^{m(p-1)+1}}{(b-a)^{m(p-1)}}. $$

Moreover, the inequality in (2.23) is strict since *u* is not a constant. This completes the proof of Theorem 2.2. □

### Remark 2.2

Inequality (2.7) improves inequality (1.7) significantly when $p>2$ and $m\geq 2$. We list the first six values of $\zeta (2n)$, $n = 1,2,\ldots, 6$, in Table 1.

**Table 1:** Values of $\zeta (2n)$

*n*	1	2	3	4	5	6
*ζ*(2*n*)	$\frac{\pi ^{2}}{6}$	$\frac{\pi ^{4}}{90}$	$\frac{\pi ^{6}}{945}$	$\frac{\pi ^{8}}{9450}$	$\frac{\pi ^{10}}{93{,}555}$	$\frac{691 \pi ^{12}}{638{,}512{,}875}$

For any $m\in \mathbb{N}$, we have $\zeta (2m)\leq \zeta (2)<2$, and then

2.24 $$\begin{aligned} \frac{\pi^{m(p-1)}}{2^{\frac{p-1}{2}}(2^{2m}-1)^{\frac{p-1}{2}}( \zeta (2m))^{\frac{p-1}{2}}} >& \frac{\pi^{m(p-1)}}{2^{(p-1)/2}(2^{2m})^{(p-1)/2}2^{(p-1)/2}} \\ =&\frac{\pi^{m(p-1)}}{2^{p-1}2^{m(p-1)}}= \biggl( \frac{\pi }{2} \biggr) ^{m(p-1)} \frac{1}{2^{p-1}} \\ =& \biggl( \frac{(\frac{\pi }{2})^{m}}{2} \biggr) ^{p-1}. \end{aligned}$$

Note that $( \frac{(\frac{\pi }{2})^{m}}{2} ) ^{p-1}>1$ for $m>\frac{\ln 2}{\ln \pi -\ln 2}\approx 1.535$ and $p>2$. Then, using (2.24), we get

$$ 2 \biggl( \frac{2}{b-a} \biggr) ^{m(p-1)}\frac{\pi^{m(p-1)}}{2^{(p-1)/2}(2^{2m}-1)^{(p-1)/2}( \zeta (2m))^{(p-1)/2}}>2 \biggl( \frac{2}{b-a} \biggr) ^{m(p-1)}. $$

So, Theorem 2.1 improves Theorem 2.1 of [1] significantly when $p>2$ and $m\geq 2$.

## Application

We give an application of the above Lyapunov inequality for an eigenvalue problem.

### Example 3.1

Let *λ* be an eigenvalue of the following problem:

$$ \textstyle\begin{cases} ( \vert u^{(m)}(t) \vert ^{p-2}u^{(m)}(t))'+\lambda r(t) \vert u(t) \vert ^{p-2}u(t)=0, \\ u^{(i)}(a)+u^{(i)}(b)=0, \quad i=0,1,2,\ldots,m, \end{cases} $$

where $r\in C([a,b],\mathbb{R})$, $m\in \mathbb{N}$, and $p>2$ is a constant. Then, from Theorem 2.1, we have

$$ \vert \lambda \vert >\frac{2}{\int_{a}^{b} \vert r(t) \vert \,\mathrm{d}t} \biggl( \frac{2}{b-a} \biggr) ^{m(p-1)} \frac{\pi^{m(p-1)}}{2^{(p-1)/2}(2^{2m}-1)^{(p-1)/2}(\zeta (2m))^{(p-1)/2}}. $$

## References

[1] Wang Y.Y. (2012). Lyapunov-type inequalities for certain higher order differential equations with anti-periodic boundary conditions. Appl. Math. Lett..

[2] Lyapunov A.M. (1907). Probleme général de la stabilité du mouvement (French translation of a Russian paper dated 1893). Ann. Fac. Sci. Univ. Toulouse.

[3] Cheng S.S. (1991). Lyapunov inequalities for differential and difference equations. Fasc. Math..

[4] Eliason S.B. (1974). Lyapunov inequalities and bounds on solutions of certain second order equations. Can. Math. Bull..

[5] Guseinov G.S., Kaymakcalan B. (2003). Lyapunov inequalities for discrete linear Hamiltonian systems. Comput. Math. Appl..

[6] Tang X.-H., Zhang M. (2012). Lyapunov inequalities and stability for linear Hamiltonian systems. J. Differ. Equ..

[7] Lee C., Yeh C., Hong C., Agarwal R.P. (2004). Lyapunov and Wirtinger inequalities. Appl. Math. Lett..

[8] Pachpatte B.G. (1997). Lyapunov type integral inequalities for certain differential equations. Georgian Math. J..

[9] Tiryaki A., Unal M., Çakmak D. (2007). Lyapunov-type inequalities for nonlinear systems. J. Math. Anal. Appl..

[10] Yang X.J. (2003). On inequalities of Lyapunov-type. Appl. Math. Comput..

[11] Zhang L.H., Zheng Z.W. (2017). Lyapunov type inequalities for the Riemann–Liouville fractional differential equations of higher order. Adv. Differ. Equ..

[12] Jleli M., Kirane M., Samet B. (2017). Lyapunov-type inequalities for a fractional p-Laplacian system. Fract. Calc. Appl. Anal..

[13] Çakmak D. (2010). Lyapunov-type integral inequalities for certain higher order differential equations. Appl. Math. Comput..

[14] Watanabe K., Yamagishi H., Kametaka Y. (2011). Riemann zeta function and Lyapunov-type inequalities for certain higher order differential equations. Appl. Math. Comput..

[15] Yang X.J., Lo K.M. (2014). Lyapunov-type inequalities for a class of higher-order linear differential equations with anti-periodic boundary conditions. Appl. Math. Lett..

[16] Jleli M., Nieto J.J., Samet B. (2017). Lyapunov-type inequalities for a higher order fractional differential equation with fractional integral boundary conditions. Electron. J. Qual. Theory Differ. Equ..

[17] Liu H.D. (2018). Lyapunov-type inequalities for certain higher-order difference equations with mixed non-linearities. Adv. Differ. Equ..

[18] Liu H.D., Meng F.W. (2018). Nonlinear retarded integral inequalities on time scales and their applications. J. Math. Inequal..

[19] Liu H.D., Meng F.W. (2016). Some new generalized Volterra–Fredholm type discrete fractional sum inequalities and their applications. J. Inequal. Appl..

[20] Feng Q.H., Meng F.W., Fu B.S. (2013). Some new generalized Volterra-Fredholm type finite difference inequalities involving four iterated sums. Appl. Math. Comput..

[21] Meng F.W., Shao J. (2013). Some new Volterra–Fredholm type dynamic integral inequalities on time scales. Appl. Math. Comput..

[22] Gu J., Meng F.W. (2014). Some new nonlinear Volterra–Fredholm type dynamic integral inequalities on time scales. Appl. Math. Comput..

[23] Liu H.D. (2017). A class of retarded Volterra–Fredholm type integral inequalities on time scales and their applications. J. Inequal. Appl..

[24] Cabrera I., Sadarangani K., Samet B. (2017). Hartman–Wintner-type inequalities for a class of nonlocal fractional boundary value problems. Math. Methods Appl. Sci..

[25] Liu H.D. (2018). Some new integral inequalities with mixed nonlinearities for discontinuous functions. Adv. Differ. Equ..

[26] Zhao D.L., Yuan S.L., Liu H.D. (2018). Random periodic solution for a stochastic SIS epidemic model with constant population size. Adv. Differ. Equ..

[27] Tunç E., Liu H.D. (2018). Oscillatory behavior for second-order damped differential equation with nonlinearities including Riemann–Stieltjes integrals. Electron. J. Differ. Equ..

[28] Liu H.D., Meng F.W. (2016). Interval oscillation criteria for second-order nonlinear forced differential equations involving variable exponent. Adv. Differ. Equ..

[29] Wang J.F., Meng F.W., Gu J. (2017). Estimates on some power nonlinear Volterra–Fredholm type dynamic integral inequalities on time scales. Adv. Differ. Equ..

[30] Xu R. (2017). Some new nonlinear weakly singular integral inequalities and their applications. J. Math. Inequal..

